# Tour Leaders’ Knowledge of and Attitudes toward Rabies Vaccination, Taiwan

**DOI:** 10.3201/eid2001.130673

**Published:** 2014-01

**Authors:** Chiao-Yu Huang, Hsien-Liang Huang, Shao-Yi Cheng, Chia-Wen Lu, Long-Teng Lee, Tai-Yuan Chiu, Kuo-Chin Huang

**Affiliations:** National Taiwan University Hospital, Taipei City, Taiwan (C.-Y. Huang, H.-L. Huang, S.-Y. Cheng, C.-W. Lu, L.-T. Lee, T.-Y. Chiu, K.-C. Huang);; Cardinal Tien Hospital, New Taipei City, Taiwan (H.-L. Huang);; Fu-Jen Catholic University, New Taipei City (H.-L. Huang);; China Medical University, Taichung, Taiwan (K.-C. Huang)

**Keywords:** rabies, vaccination, questionnaires, travel medicine, tour leaders, viruses, Taiwan

**To the Editor:** Tour leaders accompany and care for the health, comfort, and safety of travelers in group tours, which remain a popular method of international travel in Asian countries, including Taiwan (*1*). In addition to travel agents and physicians, tour leaders can also play a key role in the prevention and management of travel-related infectious diseases during group tours.

Rabies is a viral, vaccine-preventable, zoonotic, infectious disease that occurs throughout the world; it is almost always fatal (*2**, **3*). According to records of postexposure prophylaxis, ≈0.4% of all travelers have experienced 1 animal (at-risk) bite per month of stay in a rabies-endemic country; in the past 10 years, at least 22 confirmed cases of rabies among travelers have been reported (*4**,**5*). Given that rabies-endemic countries include many popular tourist destinations, rabies has become one of the most serious travel-related infectious diseases (*3*). In 2011, nearly half of the 9 million travelers from Taiwan participated in group tours to Southeast Asia, a highly rabies-endemic area. Thus, tour leaders might be in a position to influence rabies risk among group travelers to high-risk destinations.

To determine tour leaders’ knowledge of and attitudes toward rabies vaccination, we conducted a cross-sectional survey among those working in international tourism in Taiwan. A self-administered questionnaire was given to 191 tour leaders who attended 6 seminars in Taiwan during May–October 2010. This questionnaire (Technical Appendix) comprised 3 sections: demographic information; attitude toward rabies vaccination; and knowledge about general rabies-related information, prevention, and postexposure management. The questionnaire was based on a literature review. Statistical analysis was performed by using SPSS for Windows 11.0 (SPSS, Chicago, IL, USA) and χ^2^ test and stepwise logistic regression analysis; p value was set at 0.05.

A total of 175 (91.6%) tour leaders completed the questionnaire. Respondent mean age (± SD) was 44.5 ± 11.8 (range 20–71) years. Among them, 58.3% were women, and 82.3% had a college degree or above. A positive attitude toward preexposure rabies vaccination was reported by >90% of tour leaders (Table). Tour leaders who intended to receive vaccination showed higher willingness to recommend vaccination to group travelers. Most (46.3%) tour leaders indicated that the main factor influencing their intention to receive vaccination was disease severity. However, the mean percentage of accurate responses to rabies-related questions was only 52.4% (Table). Most (49.1%) tour leaders incorrectly thought that it often takes 1 day to 1 week for symptoms of rabies to develop after a person is infected. Only 44.6% of respondents knew that the mortality rate for rabies is >99% after symptoms appear. Regarding the question “Where is rabies present?” the most often chosen incorrect answer was Southeast Asia and mainland China only (32.0%). A positive attitude toward rabies vaccination and poor knowledge were noted regardless of tour leader age and education level. Multiple logistic regression analyses showed that the response to the question about mortality rate was a significant predicting variable regarding tour leaders’ attitudes toward vaccination. Tour leaders who understood the high mortality rate associated with rabies tended to receive preexposure rabies vaccination (odds ratio 5.578, 95% CI 1.190–26.170, p = 0.029) and would recommend vaccination to group travelers (odds ratio 15.931, 95% CI 1.840–138.090, p = 0.012).

**Table:** Respondent’s attitude and knowledge of rabies and vaccination (n = 175), Taiwan, 2010*

Survey section, questions	Response, %					
Yes	No	No idea	Correct answer	Incorrect answer	Don’t know

Section II: attitude toward rabies vaccination						
1. Do you intend to receive rabies vaccination before visiting a rabies-endemic area?	92.6	3.4	4.0	NA	NA	NA
2. Will you recommend rabies vaccination to travelers before they visit a rabies-endemic area?	94.3	1.7	4.0	NA	NA	NA
Section III: knowledge about rabies						
1. Transmission mode	NA	NA	NA	97.1	1.7	1.1
2. Infectious agent	NA	NA	NA	77.7	16.0	6.3
3. Particular symptom	NA	NA	NA	51.4	37.7	10.9
4. Incubation period	NA	NA	NA	25.1	65.8	9.1
5. Mortality rate	NA	NA	NA	44.6	34.3	21.1
6. Rabies-endemic area	NA	NA	NA	38.3	45.1	16.6
7. Preexposure vaccination protocol	NA	NA	NA	21.7	52.1	26.2
8. Postexposure vaccination protocol	NA	NA	NA	41.7	69.7	28.0
9. Postexposure management	NA	NA	NA	73.7	14.3	12.0

*NA, not applicable.

Our study revealed that tour leaders in Taiwan had a positive attitude toward rabies vaccination but a relatively low level of knowledge about rabies. Knowledge was poor regarding clinical manifestations, rabies-endemic areas, prevention, and management. We believe that the poor knowledge reflects insufficient information or education about rabies provided to the public or to tour leaders in Taiwan, which is a rabies-free area. Previous studies revealed that most animal-bitten travelers did not receive postexposure prophylaxis consistent with World Health Organization guidelines (*4**,**6*), possibly because travelers and local health practitioners were unfamiliar with the disease (*7**,**8*). Therefore, tour leaders with adequate knowledge about rabies might be able to provide immediate information to exposed travelers.

Knowledge of the high mortality rate associated with rabies was an independent factor influencing tour leaders’ attitudes toward preexposure rabies vaccination. This finding was consistent with previous study findings that low preexposure vaccination rates among travelers might result from the lack of knowledge among the travelers themselves or among their pretravel health care providers (*5**,**9*). In recent years, the World Health Organization and the GeoSentinel Surveillance Network recommended that persons planning to visit rabies-endemic areas receive preexposure prophylaxis before traveling (*6**,**10*). Understanding the factors influencing acceptance of vaccination could help governments develop and institute strategies for disease prevention. Thus, the Taiwan government should enhance tour leaders’ knowledge about rabies, especially regarding the high mortality rate. Education of tour leaders could, in turn, increase vaccination rates and help with prevention and management of rabies.

The results of this study are relevant for countries other than Taiwan because many Asian tourists participate in group tours. We suggest that governments place more emphasis on tour leaders’ education concerning travel medicine. Such education could not only improve the quality of group tours but also help prevent travel-related infectious diseases.

Technical Appendix Questionnaire administered to tour leaders in Taiwan, in English and in Mandarin (as administered).
