# Supplementary material for: Tour Leaders’ Knowledge of and Attitudes toward Rabies Vaccination, Taiwan
Source: Emerg Infect Dis. 2014 Jan;20(1):157–9. doi: 10.3201/eid2001.130673 (PMC3884720; doi:10.3201/eid2001.130673)
Supplement: Technical Appendix — Questionnaire administered to tour leaders in Taiwan, in English and in Mandarin (as administered). [file 13-0673-Techapp-s1.pdf]

Article DOI: http://dx.doi.org/10.3201/eid2001.130673

Tour Leaders' Knowledge of and Attitudes Toward Rabies Vaccination, Taiwan

Technical Appendix

Questionnaire Survey about Rabies and Vaccination

Section I: Demographic information

Q. Age: What is your age? ____years old

Q. Gender: Are you Male or Female?

- ☐ ˙Male
- ☐ ˙Female

Q. Marital Status: What is your marital status?

- ☐ ˙Single
- ☐ ˙Married

Q. Education: What is the highest level of education you have completed?

- ☐ ˙Elementary school
- ☐ ˙Junior high school
- ☐ ˙Senior high school
- ☐ ˙College
- ☐ ˙Graduate school or above

Section II: Attitude towards rabies and vaccination

1. Do you intend to receive rabies vaccination before visiting a rabies-endemic area?
2. Will you recommend rabies vaccination to travelers before they visit a rabies-endemic area?
3. Which is the main factor influencing your intention to receive rabies pre-exposure vaccination?(Please sort it according to level of influence)
    a. Disease severity
    b. Disease prevalence
    c. Vaccine efficacy
    d. Vaccine cost

e. Adverse effect of vaccine
f. Promotion by government
g. Past experience of immunization
h. Self-assessed general health condition

Section III: Multiple choice questions

1. How is rabies transmitted?
    a. By mosquito bites;
    b. By housefly contacts
    c. Via food, Air transmission
    d. By animal bites
    e. Sexual transmission
    f. I don't know
2. What is the pathogen for rabies?
    a. Bacteria
    b. Virus
    c. Parasites
    d. I don't know
3. What is the particular symptom of rabies in human at the later stage of infection?
    a. Fever
    b. A fear of water
    c. Jaundice
    d. Cough
    e. Frequency of urine
    f. I don't know
4. How long does it often take for symptoms of rabies to develop after a person is infected?
    a. One day
    b. One day to one week
    c. One week to one month
    d. One month to three months
    e. Over three months
    f. I don't know
5. What is the mortality rate of rabies once symptoms present?
    a. Less than 5%
    b. 5% to 50%
    c. 50% to 99%

    d. Over 99%
    e. I don’t know
6. Where is rabies present?
    a. Europe
    b. Southeast Asia and Mainland China
    c. Africa
    d. South Asia and India
    e. All the regions above
    f. I don’t know
7. How many injections of rabies vaccine should be administered for the pre-exposure vaccination before travel?
    a. One dose
    b. Two doses
    c. Three doses
    d. Four doses
    e. Five doses
    f. I don’t know
8. How many injections of rabies vaccine should be administered once bitten by animals in rabies-endemic area for post-exposure prophylaxis if not previously immunized?
    a. One dose
    b. Two doses
    c. Three doses
    d. Four doses
    e. Five doses
    f. I don’t know
9. What should travelers do if they are bitten or scratched by an animal in a rabies-endemic area?
    a. Local treatment of the wound
    b. Administration of RIG(Rabies Immunoglobulin)
    c. Administration of rabies vaccine
    d. All of above
    e. I don’t know

Questionnaire Survey about Rabies and Vaccination (as administered, in Mandarin)

台大旅遊醫學教育訓練中心問卷

一、基本資料 :

1. 年齡 : ______ □
2. 性別 : □男 □女
3. 目前婚姻狀況 : □單身 □已婚
4. 教育程度 : □小學 □國中 □高中 □大學 □研究所或以上

二、狂犬病及其疫苗相關問題 :

1. 若即將要前往狂犬病流行地區 ,本身是否願意施打暴露前預防性的狂犬病疫苗?
2. 若民眾要前往狂犬病流行地區 ,是否願意建議民眾施打暴露前預防性的狂犬病疫苗?
3. 以下是可能影響您本身是否願意施打或建議民眾施打類似狂犬病疫苗等成人疫苗的相關因素 ,請
   的重要程度由影響最大排序至影響最小
   - 甲、 疾病嚴重度
   - 乙、 疾病流行率
   - 丙、 疫苗有效性
   - 丁、 疫苗花費
   - 戊、 疫苗副作用
   - 己、 政府政策有無鼓勵
   - 庚、 過去疫苗接種經驗
   - 辛、 自覺身體健康情形

三、單選題

1. 您認為狂犬病主要是如何傳染的 ?
   □蚊子叮咬 □蒼蠅 □不乾淨的食物 □飛沫 □動物咬傷
   □性交 □不知道
2. 您認為狂犬病的致病原為何 ?
   □細菌 □病毒 □寄生蟲 □不知道
3. 以下何者是狂犬病發病後期較特別的症狀 ?
   □發燒 □恐水症 □黃疸 □咳嗽 □頻尿 □不知道
4. 若得到此疾病 ,大約經過多久才會出現症狀
   □一天 □一天至一個禮拜 □一個禮拜至一個月 □一個月至三個月
   □三個月以上 □不知道

5. 若感染並出現狂犬病症狀,其致死率大約是多少?

□<5% □5-50% □50-99% □99% □不知道

6. 您認為世界哪些區域有狂犬病病例?

□歐洲 □東南亞及中國大陸 □非洲 □印度及南亞 □美洲

□以上地區都有 □不知道

7. 若為了預防狂犬病,而進行所謂的暴露前狂犬病疫苗接種,總共需接種幾劑疫苗?

□一劑 □二劑 □三劑 □四劑 □五劑 □不知道

8. 若從未接種過狂犬病疫苗,但被可能致病的動物咬傷,而要進行暴露後的狂犬病疫苗接種,總共需接種幾劑疫苗?

□一劑 □二劑 □三劑 □四劑 □五劑 □不知道

9. 若在狂犬病流行地區被動物咬傷,最好進行那些處置,以降低得病風險?

□清理並消毒傷口 □施打暴露後狂犬病疫苗 □施打狂犬病免疫球蛋白 □以上皆是不知道
